# Long Bone Histology and Growth Patterns in Ankylosaurs: Implications for Life History and Evolution

**DOI:** 10.1371/journal.pone.0068590

**Published:** 2013-07-24

**Authors:** Martina Stein, Shoji Hayashi, P. Martin Sander

**Affiliations:** Steinmann Institut für Geologie, Mineralogie und Paläontologie, Universität Bonn, Bonn, Germany; Ludwig-Maximilians-Universität München, Germany

## Abstract

The ankylosaurs are one of the major dinosaur groups and are characterized by unique body armor. Previous studies on other dinosaur taxa have revealed growth patterns, life history and evolutionary mechanisms based on their long bone histology. However, to date nothing is known about long bone histology in the Ankylosauria. This study is the first description of ankylosaurian long bone histology based on several limb elements, which were sampled from different individuals from the Ankylosauridae and Nodosauridae. The histology is compared to that of other dinosaur groups, including other Thyreophora and Sauropodomorpha. Ankylosaur long bone histology is characterized by a fibrolamellar bone architecture. The bone matrix type in ankylosaurs is closest to that of *Stegosaurus*. A distinctive mixture of woven and parallel-fibered bone together with overall poor vascularization indicates slow growth rates compared to other dinosaurian taxa. Another peculiar characteristic of ankylosaur bone histology is the extensive remodeling in derived North American taxa. In contrast to other taxa, ankylosaurs substitute large amounts of their primary tissue early in ontogeny. This anomaly may be linked to the late ossification of the ankylosaurian body armor. Metabolically driven remodeling processes must have liberated calcium to ossify the protective osteodermal structures in juveniles to subadult stages, which led to further remodeling due to increased mechanical loading. Abundant structural fibers observed in the primary bone and even in remodeled bone may have improved the mechanical properties of the Haversian bone.

## Introduction

In the field of dinosaur studies, the investigation of paleohistological features has become a popular and efficient method to supply information on growth patterns, life history and evolutionary mechanisms in numerous taxa (e.g. [1]–[13]). Previous studies of the ornithischian clade Thyreophora have been comparatively limited, focusing mainly on the most peculiar feature shared by this group, the body armor [14]–[22]. Insights into the growth strategies of Thyreophora have been provided by the examination of several long bones of *Scutellosaurus* [23] and a detailed study of long and girdle bone histology in *Stegosaurus* [19], [24]. However, to date absolutely nothing is known about long bone histology in the Ankylosauria.

The objective of this study is to offer a first description of ankylosaurian long bone histology based on several limb elements, which were sampled from different individuals of the two clades Ankylosauridae and Nodosauridae. Mainly for reasons of availability, the investigation focuses on material originating from the late Campanian Judith River Group of Dinosaur Provincial Park, Alberta, Canada, and from the late Campanian Two Medicine Formation of Montana, USA. Therefore, most of the histologies described here document the state in derived taxa. An exception is represented by two samples of the more basal nodosaurid *Hungarosaurus tormai,* which was collected in the Santonian of western Hungary [25]–[26].

Throughout life, primary bone tissue is continuously being replaced by new bone, in part through the formation of secondary osteons, also known as Haversian systems. Bone consisting of secondary osteons is variously known as Haversian bone, secondary bone, or osteonal bone. The explanation behind Haversian bone formation has been controversial and strongly debated over the past several decades. Several hypotheses about the role and mechanical effects of Haversian bone have been proposed: 1) removal of necrotic bone tissue [27]–[29]; 2) repair of microcracks [30]–[31]; 3) improving blood supply [32]; 4) mineral homeostasis [33]–[34]; 5) changing the grain of the bone [35]–[37]; and 6) pathology [38]. Undoubtedly, the purpose of Haversian systems cannot be explained by just one universal hypothesis. Obviously the process and initiation of remodeling represents a complex combination of different physiological and mechanical mechanisms and circumstances. However, presently, there is no satisfying consensus among researchers.

## Materials and Methods

### Materials

This study focuses on the bones of the stylopodium and zeugopodium of Ankylosauria. The majority of the sampled material was collected in the Upper Cretaceous Judith River Group of Dinosaur Provincial Park, Alberta, Canada, or the Upper Cretaceous Two Medicine Formation of Montana, USA. Information on institutional abbreviations appearing in the inventor numbers of the concerned specimens is available in the supporting material (Text S1). Generally, these North American materials comprise isolated long bones except for the humerus, radius, ulna and fibula of a single individual (TMP 1998.98.1) of *Edmontonia rugosidens* and a fibula of Ankylosauridae indet. (NSM PV20381). The North American material often was not positively identifiable to genus level, and most of it is labeled in the collections as Ankylosauridae indet. or Nodosauridae indet. In addition, the taxonomy of North American ankylosaurids is currently contentious [39]–[41]. Therefore, we refrained from assigning generic names to all North American ankylosaurid specimens in this paper, even if some of the labels were more specific. Among the nodosaurids, the individual TMP 1998.98.1 could be assigned to *Edmontonia rugosidens*.

**Figure 1:**
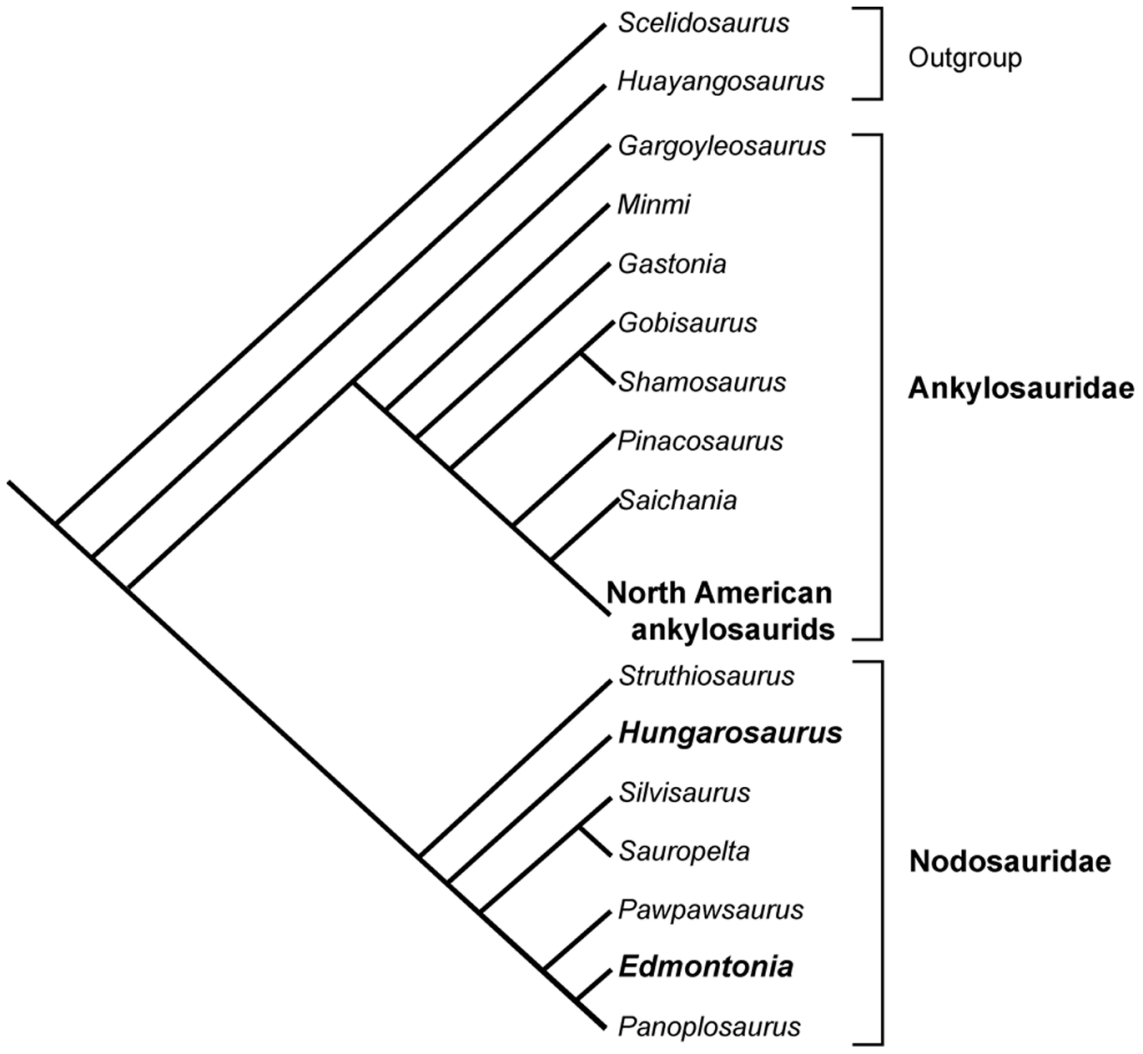
Phylogeny of the Ankylosauria based on Ősi and Makádi (2009). Studied taxa are indicated in bold.

Furthermore two bones, a femur and an ulna, of the recently described “fifth skeleton” (MTM 2007.25 [26]) of *Hungarosaurus tormai* are included in this study. This specimen was collected from the Upper Cretaceous Csehbánya Formation in the Bakony Mountains of western Hungary. Whereas Ösi and Makádi [26] found *Hungarosaurus* to represent a basal taxon among Nodosauridae (Figure 1), a more recent study recovered the taxon as a derived nodosaurid [42].

An overview of the sampled specimens with specimen numbers and measurements is given in Figure 1 and Table 1. All necessary permits were obtained for the described field studies. We obtained permissions from the various museums/institutions (i.e. NSM, MTM, TMP and ROM) to access the collections. All fossil specimens were collected by the respective museums/institutions.

**Table 1:** Sampled individuals with specimen numbers and measurements.

Family	Taxon	Specimen Nr.	Sampled Bone	Bone length(cm)	%Maximum Known Size
Ankylosauridae	Ankylosauridae indet.	ROM 47655	Humerus	45.7	92
		TMP 1997.12.220	Radius	21.5	72
		TMP 1982.16.264	Ulna	22.4	48
		TMP 1982.9.3	Femur	48.5	80
		NSM PV20381*	Fibula	26.6	65
		ROM 1930	Rib	N.A.	N.A.
Nodosauridae	*Hungarosaurus tormai*	MTM 2007.25.2	Ulna	incomplete	N.A.
		MTM 2007.25.1	Femur	incomplete	N.A.
	*Edmontonia rugosidens*	TMP 1998.98.1	Humerus	57.7	97
			Radius	41.0	
			Ulna	40.6	
			Fibula	42.2	
	Nodosauridae indet.	TMP 1981.19.15	Humerus	incomplete	N.A.
		TMP 1982.19.267	Radius	19.0	40
		TMP 2007.20.57	Ulna	48.4	100
		TMP 1981.16.434	Rib	incomplete	N.A.
		ROM 1215	Rib	incomplete	N.A.

Percentage maximum size of all specimens was calculated based on greatest length of each limb bone against the length of respective element of the largest adult ankylosaurid specimen (AMNH 5403, *“Euoplocephalus tutus*”) for ankylosaurid specimens and the largest adult Nodosaurid indet. (TMP 2007.20.57) for nodosaurid specimens. *The sample consists of the entire shaft cross section. Abbreviations: N.A., not applicable.

### Methods

With the exception of the entire shaft cross section of NSM PV20381, samples were taken by using the core-drilling method, which was established by Sander [3] in the course of his work about the Tendaguru sauropods (see also [43]). During this minimally destructive procedure, cores are taken from the narrowest part of the diaphysis. This site in the middle of the bone shaft or immediately distal to it presents the thickest and concurrently oldest cortex. Therefore, ideally almost the complete growth record is preserved. To minimize effects of shape change of the bone during growth on the growth record, cores were taken at the posterior side in the anterior limb bones and the anterior side in the posterior limb bones, respectively. In sauropods and stegosaurs, this location preserves the most complete growth record [3], [12], [24].

The coring method requires a portable coring device, where a normal household electric drill is mounted in a drill press and is equipped with a diamond-studded drill bit of 1.6 cm in diameter. For drilling, the bones were placed on the foot of the drill press, and sand bags were used to stabilize the bones. During the coring process, a circular plasticine dam filled with water as lubricant prevented the drill bit from overheating. Where possible, cores were drilled through the entire bone shaft, sampling the medullary region and the opposite cortex as well.

The cores were embedded in polyester resin (Araldite) and cut longitudinally in the cross-sectional plane of the bone. Because of the high microporosity of the samples and to reduce the risk of air bubbles, the freshly cut surfaces were impregnated again with resin and placed in a vacuum chamber. Sectioned surfaces were smoothed with grinding powder and afterwards glued onto a glass slide with Araldite and ground to a thickness of about 60 to 100 µm. Finally a glass cover slip for sample protection and increasing optical properties was used. Finished sections were examined with standard light microscopic techniques in plane-polarized transmitted light and cross-polarized light using a Leica DMLP Polarizing Microscope configured with a 360° rotating stage and PL filters and using Leica N PLAN 40/0.65, N PLAN POL 10/0.25, and N PLAN POL 2.5/0.07 objective lenses. Plane- and cross-polarized light images were acquired with a Leica DFC420 color camera using the Leica ImageAccess easyLab 7 software (Leica, Wetzlar, Germany).

Percentage maximum size of all specimens was calculated based on greatest length of each of their limb bone compared to the length of the respective element of the largest known Campanian specimen. This was the individual AMNH 5403 of *Euoplocephalus tutus* for the ankylosaurid specimens and the Nodosauridae indet. individual (TMP 2007.20.57) for all of the nodosaurid specimens except the *Hungarosaurus* specimens. Since AMNH 5403 has only a humerus preserved, a predictive regression equation of the length of the radius, ulna, femur, and fibula against the humerus was calculated from two nearly complete skeletons of North American Campanian ankylosaurids, traditionally identified as *Euoplocephalus* (AMNH 5404 and NSM PV20381). Similarly, because the largest Campanian nodosaurid (TMP 2007.20.57) preserves only an ulna, a predictive regression equation of the length of the humerus, radius and fibula against the ulna was calculated from several nodosaurid skeletons (AMNH 5665, HMNS 11, CMN 8531, ROM 1215 and TMP 1998.98.1). The relative sizes of several individuals could not be determined because their remains are too fragmentary. These include the *Hungarosaurus* specimens (MTM 2007. 25), the ankylosaurid ribs (ROM 1930), and the nodosaurid humerus (TMP 1981.19.15) and ribs (ROM 1215 and TMP 1981.16.434).

The histological terminology used in this study follows that reviewed by Francillon-Vieillot et al. [44]. The terminology for life history and life history events is based on the plesiomorphic amniote life history in which sexual maturity occurs well before full size (i.e. the growth asymptote) is reached. This is the pattern hypothesized for other non-avian dinosaurs as well (e.g. [3], [9], [12], [34], [45]) and is reflected in a distinct slow-down in linear growth well before full size is reached. This leads to the following definitions for the purpose of this paper: a juvenile is an animal that has not yet reached sexual maturity, a subadult is an animal that has not yet reached sexual maturity but is close to it (see also [19]), and an adult is an animal that has reached sexual maturity but not necessarily full size.

## Results

### Ankylosauridae

#### Ankylosauridae indet

**Figure 2:**
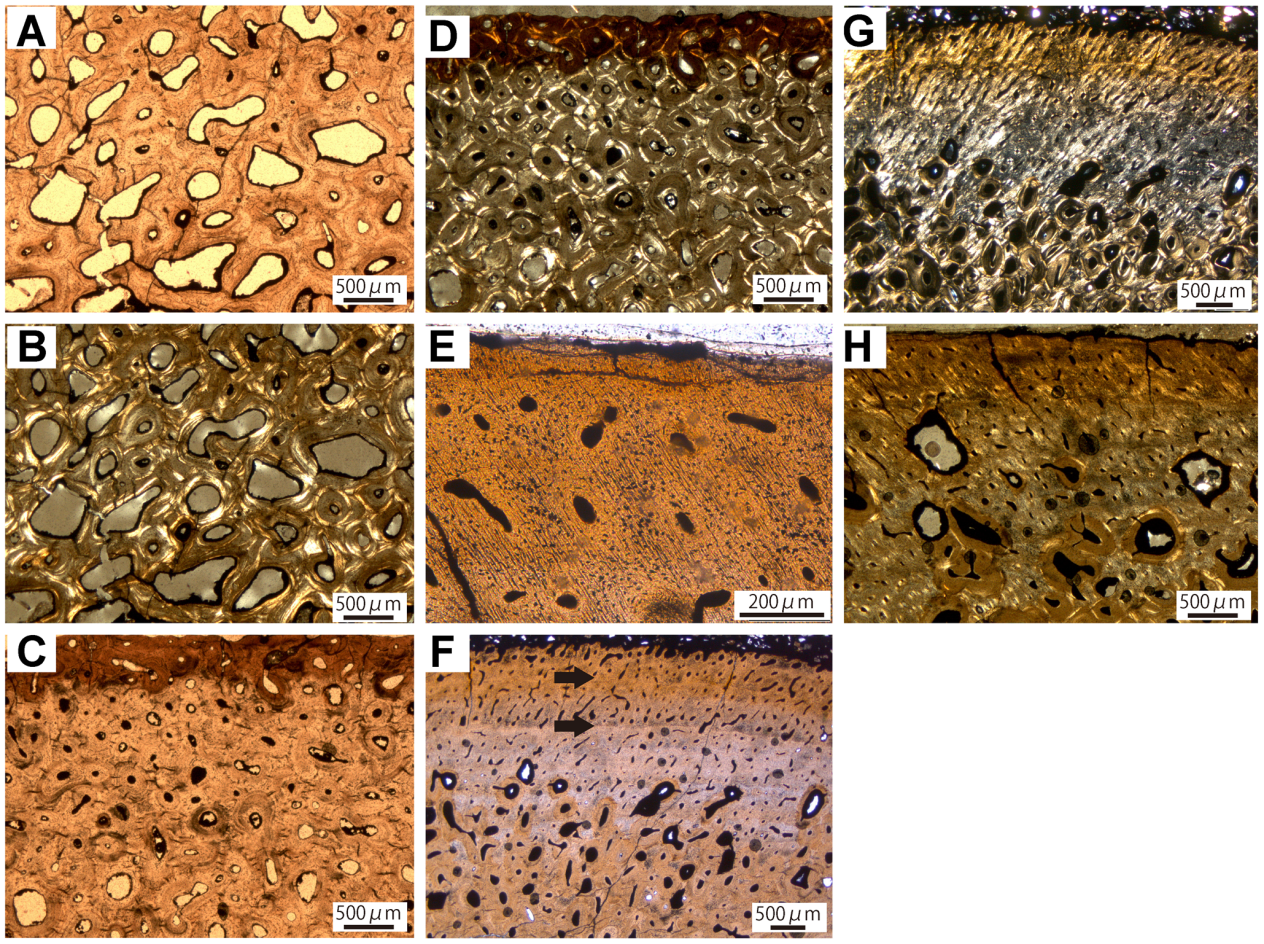
Long bone histology of Ankylosauridae indet. A, Inner cortex of the humerus (ROM 47655) showing secondary osteons of different shapes and stages of infilling. B, Same view in cross-polarized light. C, Heavily remodeled outer cortex of the radius (TMP 1997.12.220). D, Same view in cross-polarized light. E, Anterior outer cortex of the ulna (TMP 1982.16.264) displaying obliquely oriented unmineralized Sharpey’s fibers in primary bone tissue with primary osteons. F, Middle and outer posterior cortex of the ulna (TMP 1982.16.264) showing transition from dense Haversian tissue to predominantly primary bone with black arrows marking “bright lines” in outer cortex; Vascular canals opening to the surface indicate an actively growing individual. G, Same view in cross-polarized light. H, Anterior cortex of the ulna (TMP 1982.16.264) with active resorption cavities in outer cortex showing a generally stronger remodeling than the histology of the posterior cortex.

The only sampled ankylosaurid humerus, ROM 47655, is from a large individual (92% maximum size; Table 1). The core comprises the posterior cortex and large parts of the medullary region. The preserved trabeculae, which are generally uncrushed, build up a massive network of trabecular bone, producing a comparatively compact medullary region, and grade into the compact bone of the inner cortex. Dense Haversian bone dominates the cortical region with at least three generations of secondary osteons present in the inner and two generations in the outer parts. Resorption cavities without infill, representing actively proceeding cutting cones, are abundant in the perimedullary to inner cortex. The new Haversian canals, as well as the more mature secondary osteons, are variable in shape and size (Figure 2A–B). Therefore, the secondary bone develops a heterogeneous appearance. Canals are linked or fused, and in places secondary osteons appear as one large structure and can have multiple canals, creating a reticular vascular pattern. A remarkable feature is the presence of diffusely oriented structural fibers (*sensu* [16]) within the dense Haversian bone of the cortex. They tend to occur in bundles, imprinting and blurring the original texture of the bone. Osteocyte lacunae are generally round and flattened along the lamellae of the secondary osteons. In the outermost cortex a belt of primary bone is present. It includes woven-fibered bone tissue with primary osteons. The vascular canals are oriented longitudinally and their diameter is small (30–40 µm). The cortical bone tissue is composed of fibrolamellar tissue, made up of a matrix showing a mixture of woven and parallel-fibered tissue. Common features observed in all thin sections of the Dinosaur Provincial Park material are radial cracks in the periphery of secondary osteons, which are caused by early diagenetic desiccation [46].

The sampled radius, TMP 1997.12.220, is from an individual of 72% of maximum size, which could not be assigned to species (Table 1). The core comprises the posterior and anterior cortex as well as the medullary region. The medullary region of TMP 1997.12.220 consists of a moderately dense network of well-developed trabeculae. Within the lamellar tissue of the trabeculae, abundant structural fibers are predominantly arranged in bundles. Towards the inner cortex the tissue grades into dense Haversian bone, which is overprinted by structural fibers. These fibers generally occur in bundles and cause a fraying out effect of the secondary osteons. This phenomenon makes it difficult to distinguish the several generations of osteons, because the structural fibers blur the typical resorption lines. However, several generations of secondary osteons dominate the tissue up to the outermost cortex with resorption cavities in different stages of infilling. Several Volkmann’s canals, which link different Haversian systems, create a reticular vascular pattern. The osteocyte lacunae generally have a rounded, regularly shaped appearance; only in some parts they are round. Because of the extensive remodeling of the whole cortex (Figure 2C–D), primary bone is restricted to interstitial patches in the outermost cortex. These reveal fibrolamellar bone with a parallel-fibered matrix with longitudinal vascular canals and abundant Sharpey’s fibers.

The only sampled ankylosaurid ulna, TMP 1982.16.264, is from a presumably juvenile individual of 48% maximum size that could not be assigned to species (Table 1). The core comprises the posterior and anterior cortex as well as the medullary region. The medullary region of TMP 1982.16.264 displays a network of densely arranged, well-developed trabeculae, which enclose the medullary and perimedullary cavities. Structural fibers are present, but are sparse compared to what is seen in other ankylosaurid elements. The osteocyte lacunae within the trabecular bone are round and irregularly shaped. In the inner cortex, much of the primary tissue is substituted by two generations of secondary osteons, which have varying shapes and sizes. Active remodeling is indicated by the presence of resorption cavities. Haversian canals are fused or elongated, producing a reticular vascular pattern. Osteocyte lacunae are visible as rounded dots distributed along the osteonal lamellae. The primary tissue, which is well preserved in the inner parts of the cortex, is easy to identify by the presence of obliquely oriented Sharpey’s fibers (Figure 2E), which imprint the fabric of all primary structures, including the fibers of the bone matrix and vascular canals. Vascularization is high compared to most of the other specimen sampled, with canals of the primary osteons showing a reticular pattern (Figure 2F–H). The presence of vascular canals, which open to the bone surface, indicates active growth for the individual at the time of death (Figure 2F–H). The primary tissue is densely packed with round osteocyte lacunae. In cross-polarized light, there are bright lines in the posterior part of the cortex (Figure 2G). They are not fully developed lines of arrested growth (LAGs), but nevertheless indicate a cyclical growth pattern, because their appearance is followed by an accumulation of osteocyte lacunae, indicating strong cell activity, and a zone of enhanced vascularity. The remodeling of the inner cortex abruptly decreases in the middle cortex, indicating a progressive remodeling front. This pattern is visible in the posterior and anterior cortex with the notable difference that the remodeling in the anterior cortex is more advanced towards the external part of the cortex (Figure 2G–H).

**Figure 3:**
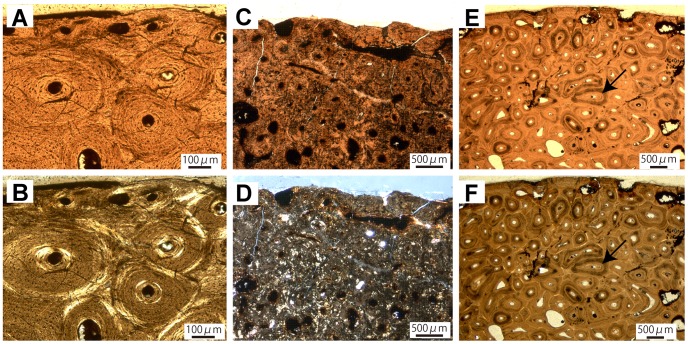
Long bone histology of Ankylosauridae indet. A, Outer cortex of the femur (TMP 1982.9.3) with few longitudinally arranged primary osteons in outermost cortex. B, Same view in cross-polarized light. C, Outer cortex of the fibula (NSM PV20381) showing fibrolameller bone with heavily remodeling. D, Same view in cross-polarized light. E, Heavily remodeled cortex of the rib (ROM 1930). Dark shades in secondary osteons (see arrows) are bundles of structural fibers blurring the crystallite orientation. F, Same view in cross-polarized light.

The sampled femur TMP 1982.9.3 is from a relatively large individual, which is 80% maximum size (Table 1). The core only comprises the anterior cortex and parts of the medullary region, which is largely crushed. The collapsed trabeculae contain many structural fibers, which are generally oriented circumferentially around the perimedullary cavities. Osteocyte lacunae abound throughout the whole cortex. In the trabecular bone and the inner cortex, they are flattened and elongated in regions of structural fiber accumulation. Otherwise, their appearance is generally round. The cortex is extensively remodeled into dense Haversian bone, comprising at least three generations of secondary osteons with osteons of older generations generally being larger than younger ones. In the inner parts of the cortical region, the shape and size of the Haversian systems are variable. Towards the outer regions the Haversian bone has a more uniform appearance. Structural fibers are abundant throughout the whole cortex, but while the structural fibers present in the inner cortex match the appearance of those in the trabeculae, their orientation becomes more diffuse towards the outer cortex. The same goes for the osteocyte lacunae; in the inner cortex they are elongated in response to the structural fiber orientation. In the outer cortex they are round, irregularly shaped structures. In the outermost cortex some primary bone is preserved. The tissue is predominantly woven-fibered with subordinate parallel-fibered parts (Figure 3A–B). The vascularity is high compared to most of the other specimen sampled, with several longitudinal vascular canals developed as primary osteons.

The sampled fibula (NSM PV20381) is from a skeleton of 65% maximum size (Table 1). The histological sample is a complete cross section, showing the entire cortex and the medullary region. The trabecular bone in the medullary region of the fibula is similar to that of the other limb bone elements. The abundant structural fibers occur in bundles and are accompanied by flattened and elongate osteocyte lacunae. The cortex is heavily remodeled into dense Haversian bone. In the outer part of the cortex, some primary bone consisting of a tissue intermediate between fibrolamellar and parallel-fibered bone is preserved (Figure 3C–D).

The histology of the ribs of ROM 1930, an individual of *Euoplocephalus* (Table 1), shows predominantly dense Haversian tissue. The texture of the secondary osteons is overprinted by abundant structural fibers (Figure 3E–F). Both ribs display some primary tissue with few simple vascular canals embedded in a mixture of woven and parallel-fibred bone. The number of rounded osteocyte lacunae is comparatively low.

### Nodosauridae

#### Hungarosaurus

All histological samples of *Hungarosaurus* were taken from a single individual (MTM 2007.25; Table 1). There are no major histological differences between the ulna (MTM 2007.25.2) and the femur (MTM 2007.25.1) of the individual.

**Figure 4:**
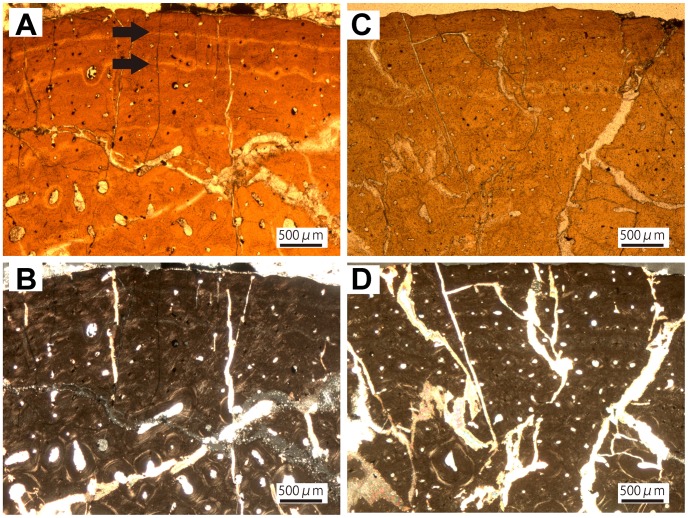
Long bone histology of the nodosaurid *Hungarosaurus tarmai.* A, Histology of the ulna (MTM 2007.25.2) with primary tissue dominating the outer cortex; Black arrows mark annulus-like modulations. B, Same view in cross-polarized light. C, Cortex of the femur (MTM 2007.25.1) revealing predominately primary bone tissue with longitudinal primary osteons. D, Same view in cross-polarized light.

The sampled ulna (MTM 2007.25.2) only comprises the posterior cortex (Table 1). The drilling was not deep enough to reach the medullary cavity but only the trabeculae of the medullary region, which are generally crushed. Primary bone generally dominates the cortical region, especially the middle and outer parts. Nevertheless, longitudinally oriented secondary osteons are abundant in the inner cortex. The other specimens develop a reticular vascular architecture in the remodeled bone of their inner to middle cortices due to the formation of secondary osteons in varying shapes and sizes. Structural fibers are absent in the Haversian bone, in contrast to the North American nodosaurids we examined. The primary cortical bone consists of fibrolamellar. The specimen shows a predominantly woven bone matrix with some parallel-fibered bone (Figure 4A–B). The strictly longitudinal vascularization is high compared to most of the other specimen sampled, and canals, opening to the bone surface, indicate a still actively growing individual. In places, obliquely oriented Sharpey’s fibers are present. Two bright lines in the outer cortex are notable (Figure 4A). Furthermore, the tissue surrounding the bright lines is characterized by a zone of longitudinal primary osteons arranged circumferentially. However, whereas these features indicate a cyclical pattern of bone deposition, they are more accurately called modulations, not true annuli. The osteocyte lacunae are generally round throughout the whole cortex in the specimen.

The sampled femur (MTM 2007.25.1; Table 1) only comprises the anterior cortical region. Because the medullary cavity is not preserved in the drill core of MTM 2007.25.1, we can only describe the cortex. The inner and middle regions of the cortex are not extensively remodeled although as many as two generations of secondary osteons are present. In the middle cortex, secondary osteons only occur scattered; in the outer cortex they are absent (Figure 4C–D). In the inner cortex active remodeling is indicated by resorption cavities. The primary bone in the inner cortex reveals typical fibrolamellar architecture with abundant primary osteons embedded in a woven matrix (Figure 4C–D). Canal orientation is strictly longitudinal, and canals are arranged in parallel circular rows. Whereas vascularity decreases towards the outer cortex, vascular canals opening to the bone surface indicate a still actively growing individual. There is gradual change in the primary bone matrix towards the outer cortex from dominantly woven bone to increasingly parallel-fibered bone. This change in fiber orientation, combined with the decreasing vascularity indicates a slow-down in growth, possibly related to sexual maturity. A low number of round osteocyte lacunae (compared to the other ankylosaur specimens) are distributed randomly.

#### Edmontonia

The sampled bones of the specimen TMP 1998.98.1, comprising humerus, ulna, radius and fibula, are part of a single individual of the nodosaurid *Edmontonia rugosidens* (Table 1), which is 97% maximum size.

**Figure 5:**
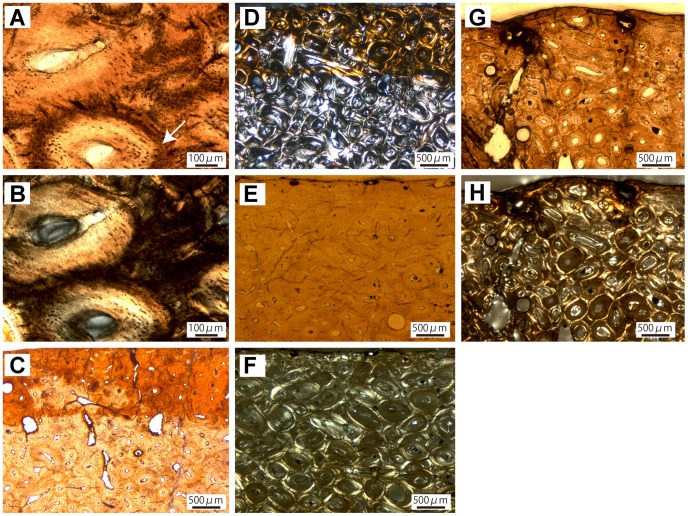
Long bone histology of the nodosaurid *Edmontonia rugosidens.* A, Inner cortex of the humerus of TMP 1998.98.1 showing secondary osteons which are strongly imprinted by unmineralized structural fibers which fray out towards the osteon margins (see a white arrow). B, Same view in cross-polarized light. C, Outer cortex of the radius of TMP 1998.98.1 showing strong remodeling and active resorption cavities. D, Same view in cross-polarized light. E, Outer cortex of the ulna of TMP 1998.98.1 with small amounts of parallel-fibered primary bone; F, Same view in cross-polarized light. G, Outer cortex of the fibula of TMP 1998.98.1 with large resorption cavities and parallel-fibered primary bone tissue. H, Same view in cross-polarized light.

In the humerus, the core sampled the posterior and anterior cortex as well as the medullary region. The medullary region of the humerus is characterized by a dense network of thick trabeculae. The lamellar tissue of the trabeculae, as well as the Haversian bone of the inner cortex, is overprinted by structural fibers resulting in frayed margins of secondary osteons, which are a common feature throughout the whole cortex. Osteocyte lacunae are flattened and elongated in regions with a high accumulation of fibers, but generally they are larger and round within the secondary osteons. There is an accumulation of unmineralized structural fibers in the inner cortex (Figure 5A–B), appearing as a darker zone under plane-polarized light. Towards the outer cortex the amount of primary bone tissue gradually increases, revealing poorly vascularized tissue intermediate between parallel-fibered and fibrolamellar bone.

In the radius, the whole medullary region is composed of a loose network of lamellar trabecular bone. The trabeculae are overprinted with randomly oriented structural fibers, which predominantly occur in bundles. The osteocyte lacunae are generally round, but in regions with structural fibers they are flattened. The trabecular bone of the medullary region grades into remodeled bone of the inner cortex. The inner cortex is completely remodeled, showing three generations of secondary osteons. These occur in different shapes and sizes. Due to fusion and immaturity of the secondary osteons, a reticular vascular pattern is developed. The osteocytes within the osteons are round. Primary bone tissue is restricted to interstitial patches between the secondary osteons but what is visible indicates a parallel-fibered organization of the primary tissue with poor vascularization and abundant Sharpey’s fibers. The osteocyte lacunae are more bulky and irregular in shape than in the secondary osteons, furthermore the patches of primary tissue show a larger osteocyte density. Towards the outer cortex the amount of primary bone, predominantly parallel-fibered matrix mixed with woven bone, gradually increases. Nevertheless, two generations of secondary osteons can be distinguished to the outermost cortex (Figure 5C–D). In these this region, the vascularization of the bone tissue is low and the vascular canals of the sparse primary osteons are oriented longitudinally. Sharpey’s fibers appear in obliquely oriented bundles.

In the ulna, the core comprises the posterior cortex as well as parts of the medullary region and shows a loose network of lamellar trabecular bone in the medullary region. The innermost cortex is remodeled, displaying at least three generations of secondary osteons. The specimen develops a reticular vascular architecture in the remodeled bone of the inner to middle cortices due to the formation of secondary osteons in varying shapes and sizes. The Haversian bone is dominated by randomly oriented secondary osteons. Structural fibers overprint the bone tissue. Neither centripetal lamellae nor the distinctive cementing lines of the secondary osteons are clearly recognizable and frayed margins are common. The primary cortical bone consists of fibrolamellar tissue with primary osteons. Some primary bone is preserved in the outermost cortex (Figure 5E–F), revealing predominantly parallel-fibered tissue. The few primary osteons are small (30–40 µm) and oriented longitudinally. Sharpey’s fibers are common. The osteocyte lacunae are generally round throughout the whole cortex, but are flattened in regions of structural fiber-bundles.

In the fibula, the drill core sampled the anterior cortex as well as parts of the medullary region. The trabecular bone in the medullary region of the fibula is similar to that of the ulna and radius. The abundant structural fibers generally occur in bundles and are associated with flat and elongate osteocyte lacunae in these regions. Due to extensive remodeling, the primary bone of the cortex is almost completely substituted by at least three generations of secondary osteons in the inner cortex and two to three generations in the outer cortex. Ongoing remodeling is indicated by the presence of several resorption cavities, which are in early stages of infilling (Figure 5G–H). Generally the cutting cones of the secondary osteons are oriented longitudinally, resulting in a homogenous appearance of the Haversian bone. There is little primary tissue remaining in the outermost cortex. It is characterized by parallel-fibered bone, which is poorly vascularized. Osteocyte lacunae are more abundant in the primary tissue than in the secondary bone. They are shaped irregularly in contrast to the rounded ones enclosed in the secondary osteons. Sharpey’s fibers predominantly occur in bundles in the primary bone.

#### Nodosauridae indet

**Figure 6:**
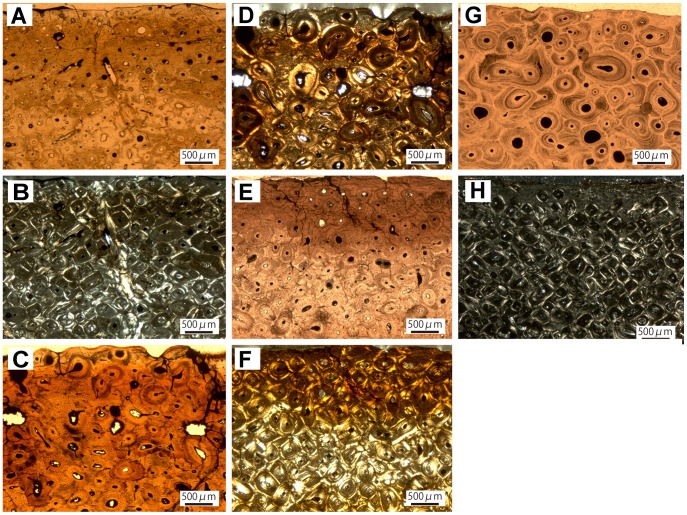
Long bone histology of Nodosauridae indet. A, Heavily remodeled outer cortex of the humerus of TMP 1981.19.15 with little amounts of parallel-fibered primary tissue. B, Same view in cross-polarized light. C, Outer cortex of the radius of TMP 1982.19.267 showing comparatively moderate remodeling. D, Same view in cross-polarized light. E, Heavily remodeled cortex of the ulna of TMP 2007.20.57. F, Same view in cross-polarized light. G, The rib of TMP 1981.16.434 with high amounts of structural fibers. H, Heavily remodeled cortex of the rib of ROM 1215 in cross-polarized light.

TMP 1981.19.15 is a fragmentary humerus that could not be assigned to species (Table 1). The core sample includes the posterior and anterior cortex plus the medullary region. The isolated secondary osteons with structural fibers are overlain by osteons of a younger generation. There is an increase in density of structural fibers in the middle and outer cortex. The secondary osteons are of varying shapes and sizes. Generally, they are fused into radially oriented structures with multiple canals in the center. The whole cortex is remodeled, the outermost cortex exhibiting two generations of secondary osteons. The primary tissue retained in the outer cortex (Figure 6A–B) is of the parallel-fibered type with round, randomly distributed osteocyte lacunae. The inner cortex of TMP 1981.19.15 is similar to TMP 1998.98.1. There is an accumulation of unmineralized structural fibers. Towards the outer cortex the amount of primary bone tissue gradually increases, revealing poorly vascularized tissue intermediate between parallel-fibered and fibrolamellar bone.

The sampled radius (TMP 1982.19.267) belongs to a small nodosaurid individual (40% maximum size; Table 1). Both cores cover the posterior and anterior cortex as well as the medullary region. The whole medullary region is composed of a loose network of lamellar trabecular bone. The trabeculae are overprinted with diffusely oriented structural fibers, which predominantly occur in bundles. The osteocyte lacunae are generally round, but in regions with structural fibers they are flattened. The trabecular bone of the medullary region grades into remodeled bone of the inner cortex. The remodeling of the cortex is moderate to the other nodosaurid specimens (Figure 6C–D), suggesting an earlier ontogenetic stage than other individuals we examined, with two generations of secondary osteons in the inner part of the cortex. The younger generation of Haversian systems seems to be generally immature. Abundant structural fibers induce a fraying out of the osteons. The primary bone, which is preserved throughout the whole cortex, shows a mixture of woven and parallel-fibered tissue with abundant Sharpey’s fibers. The vascular pattern is reticular to longitudinal with sparse canals. Osteocyte density is relatively high to other specimens with round lacunae.

The ulna, TMP 2007.20.57, is from the largest nodosaurid individual we examined (Table 1). The core comprises the posterior cortex as well as parts of the medullary region and shows a loose network of lamellar trabecular bone in the medullary region. The innermost cortex is remodeled, displaying at least three generations of secondary osteons. This specimen has a reticular vascular architecture in the remodeled bone of the inner to middle cortices due to the formation of secondary osteons in varying shapes and sizes. The abundance of structural fibers is lower than in other ankylosaur specimens. Neither centripetal lamellae nor the distinctive cementing lines of the secondary osteons are clearly recognizable and frayed margins are common. The primary cortical bone consists of fibrolamellar tissue with primary osteons. The primary tissue is restricted to isolated patches within the dense Haversian bone. Three generations of secondary osteons dominate the cortex to the outermost region of the cortex (Figure 6E–F). What is left of the primary bone shows the distinctive mixture of woven and parallel-fibered bone with a high amount of woven matrix. The osteocyte lacunae are generally round throughout the whole cortex.

In a sampled fragmentary rib (TMP 1981.16.434), the bone histology is similar to that of ROM 1215, a remodeled tissue with at least three generations of secondary osteons throughout the whole cortex up to the periosteal surface. Structural fibers are an abundant feature in the rib (Figure 6G). Patches of primary tissue reveal a mixture of woven and parallel-fibred bone with longitudinally oriented primary osteons. The collagen fibers of the primary tissue are predominantly arranged in parallel. Osteocyte lacunae are round throughout the whole cortex.

In a rib of Nodosauridae indet. (ROM 1215), the bone histology exhibits remodeled tissue with at least three generations of secondary osteons throughout the whole cortex up to the periosteal surface. Structural fibers are an abundant feature in both ribs (Figure 6H). Patches of primary tissue reveal a mixture of woven and parallel-fibred bone with longitudinally oriented primary osteons. The collagen fibers of the primary tissue are predominantly arranged in a woven pattern. Osteocyte lacunae are round throughout the whole cortex.

## Discussion

### Taxonomic Variation

In principal, the primary long bone histology of all examined specimens can be summarized as a distinctive mixture of woven and parallel-fibered tissue in a poorly vascularized matrix compared to other dinosaurian taxa. However, a few variations between the Ankylosauridae and the Nodosauridae could be taxonomic characteristics of the different families.

Generally, the long bones of the North American Ankylosauridae exhibit higher amounts of woven tissue than those of the North American Nodosauridae, even in late stages of ontogeny. For example ROM 47655, a humerus of an indeterminate ankylosaurid, histologically represents a fully-grown individual with an extensively remodeled cortex and an almost avascular zone of primary tissue in the outermost cortex. Furthermore, at 92% maximum size, it is one of the largest humeri reported in the literature to date (see also [47]–[48]) and therefore it is likely to pertain to an adult individual. Nevertheless, the amount of randomly arranged crystallites in this thin outer region is higher than in the outer cortex of the presumably younger Nodosauridae indet. (TMP 1981.19.15), which exhibits predominantly parallel-fibered tissue, not to mention the largely parallel-fibered cortices in the various bones of the adult individual of the nodosaurid *Edmontonia* (TMP 1998.98.1; Figure 5). This suggests generally higher growth rates in the Ankylosauridae, whereas nodosaurids seem to grow at slower rates, similar to those of *Stegosaurus*, as suggested by the higher amounts of parallel-fibered tissue [24].

Another taxonomic difference between Ankylosauridae and Nodosauridae is the tendency for stronger remodeling in the Ankylosauridae. Although the ankylosaurid samples represent bones from individuals of varying sizes, indicating different ontogenetic stages, the only specimen without dense Haversian tissue, but with at least two generations of secondary osteons distributed in its outermost cortex, is the juvenile ankylosaurid (TMP 1982.16.264). In contrast, all sampled bones of the nodosaurid *Edmontonia* (TMP 1998.98.1) display comparatively high amounts of primary tissue in their outer cortices.

### Ankylosaur Growth History

One of the most tried and tested methods to reconstruct the growth and life histories of dinosaurs is the counting of growth cycles, delineated by lines of arrested growth (LAGs), polish lines, annuli and zones, or modulations. Age estimates have been produced for numerous dinosaurian taxa, including several sauropodomorphs (e.g. [2]–[3], [49]), theropods (e.g. [10], [50]–[53]), and ornithischians (e.g. [4], [54]).

In general, the growth mark record in the ankylosaurs studied by us is too poor for the construction of growth curves. In a North American ankylosaurid (TMP 1982.16.264; Figure 2F–H) and in *Hungarosaurus* (MTM 2007.25.2; Figure 4), cyclical growth is recorded by bright lines in the middle to outer cortex. In the other specimens, evidence for cyclical growth is lacking or may have been obliterated by extensive remodeling of the primary tissue.

Klein and Sander [12] established a qualitative approach to ontogenetic changes in bone histology for sauropods by introducing the concept of histological ontogenetic stages (HOS). Hayashi et al. [19] applied the HOS concept to *Stegosaurus*. This raises the question whether HOS are applicable to Ankylosauria as well. Their ontogenetic criteria, which rely heavily on features of primary bone, such as the degree of primary osteon infilling and spatial arrangement of vascular canals, are difficult to apply to ankylosaurian bone histology at present because of the strong remodeling in ankylosaurs, even in early stages of ontogeny. Nevertheless, arrangement of the studied long bones in an ontogenetic sequence based on their histology is possible.

The Ankylosauridae indet. individual TMP 1982.16.264 was an actively growing individual at the time of death, which is indicated by vascular canals, opening to the bone surface (Figure 2F–H). The specimen represents an early ontogenetic stage with a distinct dominance of woven bone up to the outer cortex, indicating a high growth rate. Another indicator is the comparatively strong vascularity represented by a large number of randomly distributed and reticular canals, relative to other ankylosaur specimens sampled. Thus, the primary tissue points to a rapidly growing individual that had not yet reached sexual maturity, let alone full size. Nevertheless, remodeling had already proceeded to the middle cortex in the posterior side of the shaft and even to the outer cortex in the generally more heavily remodeled anterior side of the shaft (Figure 2H), which in other dinosaurs would suggest a later ontogenetic stage (e.g. [4], [12]).

The degree of remodeling seems to be more advanced in ankylosaurs than in other dinosaurian taxa, at least in the more derived taxa. Except for a juvenile North American ankylosaurid specimen (TMP 1982.16.264), remodeling with several generations of secondary osteons, even in the outermost cortex, is observed in all examined ankylosaurid specimens. This is true for the nodosaurids as well, in which secondary osteons are common in the outermost cortices of all specimens regardless of their sizes.

An exception appears to be the nodosaurid *Hungarosaurus*, which was only sampled from one individual. Active growth is indicated by vascular canals opening to the bone surface and remodeling is restricted to the inner to middle cortex (Figure 4). Nevertheless, this specimen seems to represent a later histological ontogenetic stage than the juvenile North American ankylosaurid (TMP 1982.16.264). Vascularity is poor, compared to other ankylosaur specimens, with strictly longitudinal canals and bright lines. The presence of higher amounts of parallel-fibered bone suggests a slow-down in growth, indicating an early adult stage after reaching sexual maturity. Thus, in *Hungarosaurus* remodeling is still advanced compared to other dinosaurian taxa, but compared to the North American nodosaurs and ankylosaurs it is relatively underdeveloped. A peculiar characteristic of this specimen is the occurrence of secondary osteons with structural fibers, which are cropped by a Haversian system of a younger generation. The cutting cones of these younger osteons resorbed not only the circumferential lamellae of the older ones, but also the structural fibers.

Remodeling seems to occur early in ontogeny when the bone is still growing appositionally and is subject to bone drift and muscle migration so the formation of structural fibers could be analog to the formation of Sharpey’s fibers due to these processes. The fact, that these fibers are an unobserved feature in the long bones of other taxa could be due to the observation that remodeling in other taxa starts later in ontogeny when the individuals’ growth rates have decreased and their bones are not subject to bone drift or muscle migration. However, this approach does not explain the presence of structural fibers in the trabecular bone of the medullary region, but it could be a reason why accumulation of structural fibers is sometimes prominent in the inner to middle cortex, as seen in the humerus of *Edmontonia* (TMP 1998.98.1; Figure 5A–B).

### Haversian Bone in Derived Ankylosauridae and Nodosauridae

Derived North American ankylosaurs and nodosaurs share the advanced remodeling and structural fibers in Haversian bone, unlike the condition in the basal nodosaurid *Hungarosaurus*. Assuming that the phylogenetic position of *Hungarosaurus* in Ösi and Makádi [26] has been correctly identified (contra [42]), these two features must have evolved convergently. This is the point of departure for the discussion of hypothesis explaining heavy remodelling and structural fibers in Haversian bone.

Probably, the most peculiar characteristic of the derived ankylosaurs is their body armor with varying arrangements of the constituent osteoderms [55], which occur in diverse shapes and sizes throughout the dermis of the individual. An important observation may be that in juveniles the dermal ossifications are not fully developed. Several studies report that in the juveniles of the Mongolian ankylosaurid *Pinacosaurus grangeri* postcranial osteoderms are simply absent [56]–[59].

Hypotheses explaining the function(s) of Haversian tissue have been discussed (summarized in [29]) but no single explanatory factor can be identified. Remodeling is known to be controlled by metabolism and mechanical loading. It has been suggested that metabolically driven remodeling is active in areas of low strain, such as trabecular bone, whereas in the high-strain areas of the cortex it is driven by mechanical loading [60]. Trabecular bone remodeling is possibly an important mechanism, because it can flexibly provide metabolic calcium for mineral homeostasis.

The simple correlation between structural fibers in both primary and secondary bone and the development of the dermal skeleton in ankylosaurs begs the question of a possible link. Ankylosaurs may have initiated remodeling cycles to mobilize minerals for osteoderm mineralization in the late juvenile stage, similar to mobilization of calcium from the skeleton in egg-laying alligators [61]. The heavy remodeling may have been caused by an asynchrononicity between limb bone growth and dermal armor development, first leading to early resorption activity in the limb bones, then to osteoderm formation, and then to increased remodeling of the limb bones. This hypothesis would predict that at some stage in life history, limb bones would show (temporary) osteoporosis. However, our small sample may not include such an individual. If this hypothesis is correct, stronger remodeling in Ankylosauridae compared to Nodosauridae might be explained by the growth of the tail club because only Ankylosauridae possess this structure (e.g. [62]), that would have needed a large amount of calcium for its formation. Osteoderm formation in the late juvenile stage in turn would have increased the weight of the animal. Although the absolute mass increase may not have been substantial, because osteoderms are generally lightweight structures (see [16], [20]), the weight gain nevertheless could have been sufficient to initiate further remodeling in a feedback loop, this time due to increased mechanical loading on the long bone.

Structural fibers are generally a feature of primary bone but their occurrence in the secondary bone of ankylosaurs has been observed before, in the osteoderms [16], [20]. Scheyer and Sander [16] suggested that these fibers served to strengthen the osteodermal bone. Since Haversian bone is known to impair the mechanical properties of bone by increasing brittleness [29], and therefore increasing the vulnerability to fatigue damage, the structural fibers could have acted as a buffer to these forces. Testing this hypothesis is difficult because structural fibers in secondary bone are unique to derived ankylosaurs so far.

Another characteristic of the remodeling pattern in ankylosaurs are the peculiar shapes and sizes of the secondary osteons. Unlike in normal secondary osteons seen in many dinosaurs and mammals, resorption cavities are rarely strictly longitudinal and completely infilled. Furthermore the osteocyte lacunae in the centripetal lamella filling in the vascular canals are not flattened as in normal secondary osteons but round in shape. These observations would suggest an unusually rapid deposition of the secondary tissue. Occurrence of this feature in other taxa is lacking.

### Comparison with Other Thyreophora

#### Scutellosaurus

The primary long bone histology of two individuals of the basal thyreophoran *Scutellosaurus* has been described by Padian et al. [23] as generally parallel-fibered bone with comparatively poor vascularization. Woven bone associated with primary osteons, characteristic for the fibrolamellar complex, is present only in the femur of the larger specimen (UCMP 170829) and in the innermost cortex of the tibia of the smaller specimens (UCMP 130580).

Some features described in *Scutellosaurus* are similar to those in ankylosaur long bone histology. Padian et al. [23] observed a decrease in vascularization towards the outer cortex without vascularity being higher in the inner cortex. Furthermore, the vascular canals tend to become smaller in diameter. These results are similar in ankylosaurs, which also show a comparatively poor vascularization of the cortical bone. The parallel longitudinal primary osteons are another feature shared by *Scutellosaurus* and ankylosaurs, particularly by *Hungarosaurus*.

Although ankylosaur long bones do show a fibrolamellar architecture, it is different from the typical fibrolamellar bone of other sampled dinosaur taxa. The ankylosaur fibrolamellar tissue is made up of a distinctive mixture of woven and parallel-fibered bone associated with predominantly longitudinal primary osteons. Therefore, the primary bone tissue as a whole largely resembles that of *Scutellosaurus*.

#### Stegosaurus

In the studies of Hayashi et al. [19] and Redelstorff and Sander [24], *Stegosaurus* bone histology is described as fibrolamellar tissue, showing a mixture of woven and parallel-fibered bone combined with poor vascularization. This peculiar mixture is also found in ankylosaur bone histology. Although overall heavy remodeling has generally obliterated the primary tissue of the inner cortical parts, the amount of woven bone decreases towards the outer cortex and grades into a more parallel-fibered architecture, indicating a slow-down in growth.

Another similarity between the two groups is the random distribution of primary osteons, which show a predominantly longitudinal, sometimes reticular pattern (Figure 2F–H) of vascularization in a comparatively poorly vascularized matrix. The canals are sometimes arranged in circular rows (Figures 2F–H, 4), which correlate with an increase in vascularization. This feature is restricted to the beginning of zones, indicating cyclical growth (Figure 4). The similarity in primary bone histology of *Stegosaurus* and ankylosaurs suggests relatively similar growth rates as well. Relative growth rate in ankylosaurs and *Stegosaurus* apparently was slower compared to other dinosaur taxa, but faster than in *Scutellosaurus*.

A notable difference in bone histology is seen in the pattern of remodeling which is more developed in the derived ankylosaurs than in *Stegosaurus*. Secondary osteons are frequent in *Stegosaurus* and remodeling is generally strong with formation of Haversian systems in the outermost cortex of still growing young adult *Stegosaurus* individuals (NSM PV20380 [19]; VFSMA001 and SMA0092 [24]). However, in the subadult *Stegosaurus* specimen (SMA RCR0603 [24]), secondary osteons only occur in the innermost cortex. Bone histology in *Hungarosaurus* resembles that of juvenile *Stegosaurus* in lacking Haversian systems in its outer cortex, although growth already had slowed down indicating an adult individual (Figure 4; see also [19] in *Stegosaurus* adults).

### Evolutionary Implications for Growth Rate and Body Size

Until recently, most attention for histological patterns in the Thyreophora has been paid to their osteoderms, focusing on the armor of ankylosaurs [16], [20], [58] and on stegosaur plates and spikes [15], [17], [19]–[21], [58]. The first insights in their long bone histology were provided by the studies of Padian et al. [23], describing long bone histology of *Scutellosaurus*, and by the detailed work on stegosaur long and girdle bone histology by Hayashi et al. [19] and Redelstorff and Sander [24].

Generally it can be summarized that ankylosaur long bone histology is most similar to that described in its sister clade, Stegosauria. In both taxa, relatively rapid growth throughout early ontogeny until reaching sexual maturity is indicated by the dominance of woven tissue and a fairly well vascularized bone. Then a slow-down in growth occurs, fiber arrangement gradually changes to a higher degree of organization, forming parallel-fibered bone, and the abundance and size of vascular canals decrease. Apparently, ankylosaurs, as well as stegosaurs, had higher growth rates than the basal *Scutellosaurus* with its predominantly parallel-fibered or even lamellar-zonal bone deposition [23]. Nevertheless, compared with other dinosaur taxa such as Ornithopoda (e.g. [54]), Ceratopsia (e.g. [4]), Theropoda (e.g. [10], [51]–[52]), and Sauropodomorpha (e.g. [2]–[3], [49]) whose long bone histology is characterized by a poor organization of fibers together with a high degree of vascularization, ankylosaurs in particular and thyreophorans in general grew more slowly.

Interesting new insights are given by the recent description of bone histology in the basal ornithischian *Lesothosaurus* [63]. The authors described typical fibrolamellar bone, characterized by the presence of primary osteons embedded in a woven-fibered matrix, which typifies a high growth rate. The long bone histology of a juvenile fibula (MNHN IG24; [63]) reveals a similarity to the juvenile North American ankylosaurid ulna (TMP 1982.16.264) described here. The arrangement and degree of vascularization, the woven-fibered tissue, and the density and appearance of osteocyte lacunae are similar to the histology observed in the juvenile ankylosaurid (TMP 1982.16.264). Although remodeling seems to be common in *Lesothosaurus*, comparable with remodeling patterns in prosauropods (see [49]), it is far from being as extensive as in ankylosaurs.

Thus, fast-growing fibrolamellar bone tissue seems to represent the plesiomorphic state for Ornithischia, suggesting the slow growth of Thyreophora to be apomorphic, representing heterochronic deceleration. The relatively high growth rate of the juveniles [19] indicated by the dominance of woven-fibered bone may have been adaptive in shortening the time before maturation of the bony body armor in the subadults [19], [22], [59]. At that ontogenetic stage, fast growth was simply not crucial due to the increased protection, and growth rates could decrease.

### Conclusions

The first in-depth analysis of ankylosaur long bone histology provides new insights into growth patterns and evolution of the Thyreophora. The examination of some North American Late Cretaceous taxa as well as of the basal *Hungarosaurus tormai* generally reveals a fibrolamellar bone architecture, which is characterized by the presence of primary osteons. However, the bone matrix type in ankylosaurs is closest to that previously described by Hayashi et al. [19] and Redelstorff and Sander [24] for *Stegosaurus*. A distinctive mixture of woven and parallel-fibered bone together with an overall poor vascularization, represented by predominantly longitudinal canals, indicates slow growth rates compared to other dinosaurian taxa which all show highly vascularized fibrolamellar bone consisting of well developed primary osteons set in a matrix of woven fibered bone.

However, ankylosaur bone histology differs in two respects from that of *Stegosaurus* and all other dinosaur groups, exceptions in individual taxa notwithstanding. One is the extensive remodeling pattern in the highly derived North American taxa, which is only surpassed by that seen in the dwarf sauropod *Magyarosaurus* [64]. Ankylosaurs substitute large amounts of their primary tissue early in ontogeny, as seen in the ulna of the juvenile indeterminate ankylosaurid indet. (TMP 1982.16.264). We suggest this early onset of remodelling is caused by mineralization of the ankylosaurian body armor. Metabolically driven remodeling processes must have liberated calcium to ossify the protective osteodermal structures in juvenile to adult stages, which, in turn, may have lead to further remodeling due to increased mechanical loading. Truly unique for ankylosaurs, and only seen in the derived Late Cretaceous Ankylosauridae and Nodosauridae so far, are the abundant structural fibers in the primary secondary bone. These have previously been described only in ankylosaur osteoderms by Scheyer and Sander [16] and Hayashi et al. [20]. In long bones, this may have improved the mechanical properties of the secondary bone.

## Supporting Information

Text S1 **Institutional abbreviations appearing in the inventor numbers of specimens.**
(DOC)

## References

[1] Chinsamy A (1995) Ontogenetic changes in the bone histology of the Late Jurassic ornithopod *Dryosaurus lettowvorbecki* . J Vert Pal 15: 96–104.

[2] Curry KA (1999) Ontogenetic histology of *Apatosaurus* (Dinosauria: Sauropoda): new insights on growth rates and longevity. J Vert Pal 19: 654–665.

[3] Sander PM (2000) Longbone histology of the Tendaguru sauropods: implications for growth and biology. Paleobiology 26: 466–488.

[4] Erickson GM, Tumanova TA (2000) Growth curve of *Psittacosaurus mongoliensis* OSBORN (Ceratopsia: Psittacosauridae) inferred from long bone histology. Zool J Linn Soc 130: 551–566.

[5] Padian K, Ricqlès A, Horner JR (2001) Dinosaurian growth rates and bird origins. Nature 412: 405–408. 11473307 10.1038/35086500

[6] Sander PM, Tückmantel C (2003) Bone lamina thickness, bone apposition rates, and age estimates in sauropod humeri and femora. Paläontol Zeit 76: 161–172.

[7] Sander PM, Klein N, Buffetaut E, Cuny G, Suteethorn V, et al (2004) Adaptive radiation in sauropod dinosaurs: bone histology indicates rapid evolution of giant body size through acceleration. Org Divers Evol 4: 165–173.

[8] Sander PM, Mateus O, Laven T, Knötschke N (2006) Bone histology indicates insular dwarfism in a new Late Jurassic sauropod dinosaur. Nature 441: 739–741. 16760975 10.1038/nature04633

[9] Sander PM, Klein N, Stein K, Wings O (2011) Sauropod bone histology and implications for sauropod biology. In: Klein N, Remes K, Gee CT, Sander PM, editors. Biology of the Sauropod Dinosaurs. Understanding the Life of Giants. Indiana University Press, Bloomington. 276–302.

[10] Bybee PJ, Lee AH, Lamm ET (2006) Sizing the Jurassic theropod dinosaur *Allosaurus*: assessing growth strategy and evolution of ontogenetic scaling of limbs. J Morphol 267: 347–59. 16380967 10.1002/jmor.10406

[11] Ricqlès A, Padian K, Knoll F, Horner JR (2008) On the origin of high growth rates in archosaurs and their ancient relatives: Complementary histological studies on Triassic archosauriforms and the problem of a “phylogenetic signal” in bone histology. Ann Paléontol 94: 57–76.

[12] Klein N, Sander PM (2008) Ontogenetic stages in the long bone histology of sauropod dinosaurs. Paleobiology 34: 247–263.

[13] Horner JR, Goodwin MB (2009) Extreme cranial ontogeny in the Upper Cretaceous dinosaur *Pachycephalosaurus* . PLoS One 4: e7626. 19859556 10.1371/journal.pone.0007626 PMC2762616

[14] Farlow JO, Thompson CV, Rosner DE (1976) Plates of the dinosaur *Stegosaurus*: Forced convection heat loss fins? Science 192: 1123–1125. 17748675 10.1126/science.192.4244.1123

[15] Buffrénil V, Farlow JO, Ricqlès A (1986) Growth and function of *Stegosaurus* plates: evidence from bone histology. Paleobiology 12: 459–473.

[16] Scheyer TM, Sander PM (2004) Histology of ankylosaur osteoderms: implications for systematics and function. J Vert Pal 24: 874–893.

[17] Main RP, Ricqlès A, Horner JR, Padian K (2005) The evolution and function of thyreophoran dinosaur scutes: implications for plate function in stegosaurs. Paleobiology 31: 291–314.

[18] Burns ME (2008) Taxonomic utility of ankylosaur (Dinosauria, Ornithischia) osteoderms: *Glyptodontopelta mimus* Ford 2000– A test case. J Vert Pal 28: 1102–1109.

[19] Hayashi S, Carpenter K, Suzuki D (2009) Different growth patterns between the skeleton and osteoderms of Stegosaurus (Ornithischia: Thyreophora). J Vert Pal 29: 123–131.

[20] Hayashi S, Carpenter K, Scheyer TM, Watabe M, Suzuki D (2010) Function and evolution of ankylosaur dermal armor. Acta Palaeontol Pol 55: 213–228.

[21] Farlow JO, Hayashi S, Tattersall G (2010) Internal vascularity of the dermal plates of *Stegosaurus* (Ornithischia: Thyreophora). Swiss J Geosci 103: 173–186.

[22] Hayashi S, Carpenter K, Watabe M, McWhinney L (2012) Ontogenetic histology of *Stegosaurus* plates and spikes. Palaeontol 55: 145–161.

[23] Padian K, Horner JR, Ricqlès A (2004) Growth in small dinosaurs and pterosaurs: the evolution of archosaurian growth strategies. J Vert Pal 24: 555–571.

[24] Redelstorff R, Sander PM (2009) Long and girdle bone histology of Stegosaurus: implications for growth and life history. J Vert Pal 29: 1087–1099.

[25] Ősi A (2005) *Hungarosaurus tormai,* a new ankylosaur (Dinosauria) from the Upper Cretaceous of Hungary. J Vert Pal 25: 370–383.

[26] Ősi A, Makádi L (2009) New remains of *Hungarosaurus tormai* (Ankylosauria, Dinosauria) from the Upper Cretaceous of Hungary: skeletal reconstruction and body mass estimation. Paläontol Zeit 83: 227–245.

[27] Enlow DH (1963) Principles of Bone Remodeling. Thomas, Springfield, Ill.

[28] Marotti G (1993) A new theory of bone lamellation. Calcif Tissue Int 53(1, Supplement): S47–S55. 10.1007/BF01673402 8275380

[29] Currey JD (2002) Bones: Structure and Mechanics. Princeton University Press, Princeton. 436p.

[30] Churches AE, Howlett CR (1981) The response of mature cortical bone to controlled time-varying loading. In: Cowin SC, editor. Mechanical properties of bone, AMD, Vol. 45. American Society of Mechanical Engineers, New York. 69–80.

[31] Mori S, Burr DB (1993) Increased intracortical remodeling following fatigue damage. Bone 114: 103–109. 10.1016/8756-3282(93)90235-3 8334026

[32] Currey JD (1960) Differences in the blood-supply of bone of different histological types. Q J Microsc Sci 101: 357–370.

[33] Schweitzer MH, Wittmeyer JL, Horner JR (2005) Gender-specific reproductive tissue in ratites and *Tyrannosaurus rex* . Science 308: 1456–1460. 15933198 10.1126/science.1112158

[34] Lee AH, Werning S (2008) Sexual maturity in growing dinosaurs does not fit reptilian growth models. PNAS 105: 582–587. 18195356 10.1073/pnas.0708903105 PMC2206579

[35] Enlow DH (1962) Functions of haversian systems. Am J Anat 110: 269–305. 13890323 10.1002/aja.1001100305

[36] Riggs CM, Lanyon LE, Boyde A (1993) Functional associations between collagen fibre orientation and locomotor strain direction in cortical bone of the equine radius. Anat Embryol 187: 231–238. 8470823 10.1007/BF00195760

[37] Riggs CM, Lanyon LE, Boyde A (1993) Mechanical implications of collagen fibre orientation in cortical bone of the equine radius. Anat Embryol 187: 239–248. 8470824 10.1007/BF00195761

[38] Otter MW, Qin YX, Rubin CT, McLeod KJ (1999) Does bone perfusion/reperfusion initiate bone remodeling and the stress fracture syndrome? Med Hypothese 53: 363–368. 10.1054/mehy.1998.0782 10616033

[39] Arbour VM, Burns ME, Sissons RL (2009) A redescription of the ankylosaurid dinosaur *Dyoplosaurus acutosquameus* Parks, 1924 (Ornithischia: Ankylosauria) and a revision of the genus. J Vert Pal 29: 1117–1135.

[40] Penkalski P (2013) A new ankylosaurid from the late Cretaceous Two Medicine Formation of Montana, USA. Acta Palaeontol Pol: doi:10.4202/app.2012.0125.

[41] Penkalski P, Blows WT (2013) *Scolosaurus cutleri* (Ornithischia: Ankylosauria) from the Upper Cretaceous Dinosaur Park Formation of Alberta, Canada. Can J Earth Sci 50: 171–182.

[42] Thompson RS, Parish JC, Maidment SCR, Barrett PM (2012) Phylogeny of the ankylosaurian dinosaurs (Ornithischia: Thyreophora). J Syst Pal 10: 301–312.

[43] Stein K, Sander PM (2009) Histological core drilling: a less destructive method for studying bone histology. In: Brown MA, Kane JF, Parker WG., editors. Methods In Fossil Preparation: Proceedings of the First Annual Fossil Preparation and Collections Symposium. 69–80.

[44] Francillon-Vieillot H, Buffrénil V, Castanet J, Géraudie J, Meunier FJ, et al.. (1990) Microstructure and mineralization of vertebrate skeletal tissues. In: Carter JG., editor. Skeletal Biomineralization: Patterns, Processes and Evolutionary Trends Vol. 1. Van Nostrand Reinhold, New York. 471–530.

[45] Lee AH, Huttenlocker AK, Padian K, Woodward NH (2013) Analysis of growth rates. In: Padian K, Lamm ET, editors. Bone histology of fossil tetrapods. University of California Press. 217–251.

[46] Pretzschner HU (2004) Fossilization of Haversian bone in aquatic environments. C R Palevol 3: 605–616.

[47] Burns ME, Sullivan RM (2011) A new ankylosaurid from the Upper Cretaceous Kirtland Formation, San Juan Basin, with comments on the diversity of ankylosaurids in New Mexico. Bull New Mexico Mus Nat His Sci 53: 169–178.

[48] Maidment SCR, Linton DH, Upchurch P, Barrett PM (2012) Limb-bone scaling indicates diverse stance and gait in quadrupedal ornithischian dinosaurs. PLoS ONE 7(5): e36904. 22666333 10.1371/journal.pone.0036904 PMC3358279

[49] Klein N, Sander PM (2007) Bone histology and growth of the prosauropod dinosaur *Plateosaurus engelhardti* von MEYER, 1837 from the Norian bonebeds of Trossingen (Germany) and Frick (Switzerland). Special Papers in Palaeontology 77: 169–206.

[50] Horner JR, Padian K, Ricqlès A (2001) Comparative osteohistology of some embryonic and perinatal archosaurs: developmental and behavioral implications for dinosaurs. Paleobiology 27: 39–58.

[51] Erickson GM, Curry Rogers K, Yerby SA (2001) Dinosaurian growth patterns and rapid avian growth rates. Nature 412: 429–433. 11473315 10.1038/35086558

[52] Erickson GM, Makovicky PJ, Currie PJ, Norell MA, Yerby SA, et al (2004) Gigantism and comparative life-history parameters of tyrannosaurid dinosaurs. Nature 430: 772–775. 15306807 10.1038/nature02699

[53] Chinsamy A (2005) The microstructure of dinosaur bone. The Johns Hopkins University Press, Baltimore, London. 195p.

[54] Horner JR, Ricqlès A, Padian K (2000) Long bone histology of the hadrosaurid dinosaur *Maiasaura peeblesorum*: growth dynamics and physiology based on ontogenetic series of skeletal elements. J Vert Pal 20: 115–129.

[55] Ford TL (2000) A review of ankylosaur osteoderms from New Mexico and a preliminary review of ankylosaur armor. Bull New Mexico Mus Nat His Sci 17: 157–176.

[56] Currie PJ (1991) The Sino/Canadian dinosaur expeditions 1986–1990. Geotimes April: 19–21.

[57] Currie PJ, Badamgarav D, Koppelhus EB, Sissons R, Vickaryous MK (2011) Hands, feet, and behaviour in *Pinacosauru*s (Dinosauria: Ankylosauridae). Acta Palaeontol Pol 56: 489–504.

[58] Arbour VM, Currie PJ (2011) Taphonomic filters of age groups of the ankylosaurid dinosaur *Pinacosaurus*. J Vert Pal, Program and Abstracts: 64.

[59] Hayashi S, Zhao Q, Watabe M, Carpenter K, Xu X (2012) Phylogenetic and ontogenetic variation of bone histology in thyreophoran osteoderms. J Vert Pal, Program and Abstracts: 108.

[60] Bouvier M, Hylander WL (1996) The mechanical or metabolic function of secondary osteonal bone in the monkey *Macaca fascicularis* . Arch Oral Biol 41: 941–950. 9031701 10.1016/s0003-9969(96)00047-7

[61] Schweitzer MH, Elsey RM, Dacke CG, Horner JR, Lamm ET (2007) Do egg-laying crocodilian (Alligator mississippiensis) archosaurs form medullary bone? Bone 40: 1152–1158. 17223615 10.1016/j.bone.2006.10.029

[62] Arbour VM (2009) Estimating impact forces of tail club strikes by ankylosaurid dinosaurs. PLoS ONE 4(8): e6738. 19707581 10.1371/journal.pone.0006738 PMC2726940

[63] Knoll F, Padian K, Ricqlès A (2010) Ontogenetic change and adult body size of the early ornithischian dinosaur *Lesothosaurus diagnosticus*: Implications for basal ornithischian taxonomy. Gondwana Res17: 171–179.

[64] Stein K, Csiki Z, Curry Rogers K, Weishampel DB, Redelstorff R, et al (2010) Small body size and extreme cortical bone remodeling indicate phyletic dwarfism in Magyarosaurus dacus (Sauropoda: Titanosauria). PNAS 107: 9258–9263. 20435913 10.1073/pnas.1000781107 PMC2889090

