# Supplementary material for: Long Bone Histology and Growth Patterns in Ankylosaurs: Implications for Life History and Evolution
Source: PLoS One. 2013 Jul 24;8(7):e68590. doi: 10.1371/journal.pone.0068590 (PMC3722194; doi:10.1371/journal.pone.0068590)
Supplement: Text S1 — Institutional abbreviations appearing in the inventor numbers of specimens. (DOC) [file pone.0068590.s001.doc]

**Institutional Abbreviations: NSM**, National Science Museum, Tokyo, Japan; **MNHN**, Muséum National d’Histoire Naturelle, Paris; **MTM**, Hungarian Natural History Museum, Budapest, Hungary; **ROM**, Royal Ontario Museum, Toronto, Ontario, Canada; **SMA**, Sauriermuseum Aathal near Zurich, Switzerland; **TMP**, Royal Tyrrell Museum of Paleontology, Drumheller, Alberta, Canada; **UCMP**, University of California Museum of Paleontology, Berkeley, California, USA; **VFSMA**, Verein für das Sauriermuseum Aathal, Switzerland..
